# Supplementary figures and images for: S/HIC: Robust Identification of Soft and Hard Sweeps Using Machine Learning
Source: PLoS Genet. 2016 Mar 15;12(3):e1005928. doi: 10.1371/journal.pgen.1005928 (PMC4792382; doi:10.1371/journal.pgen.1005928)

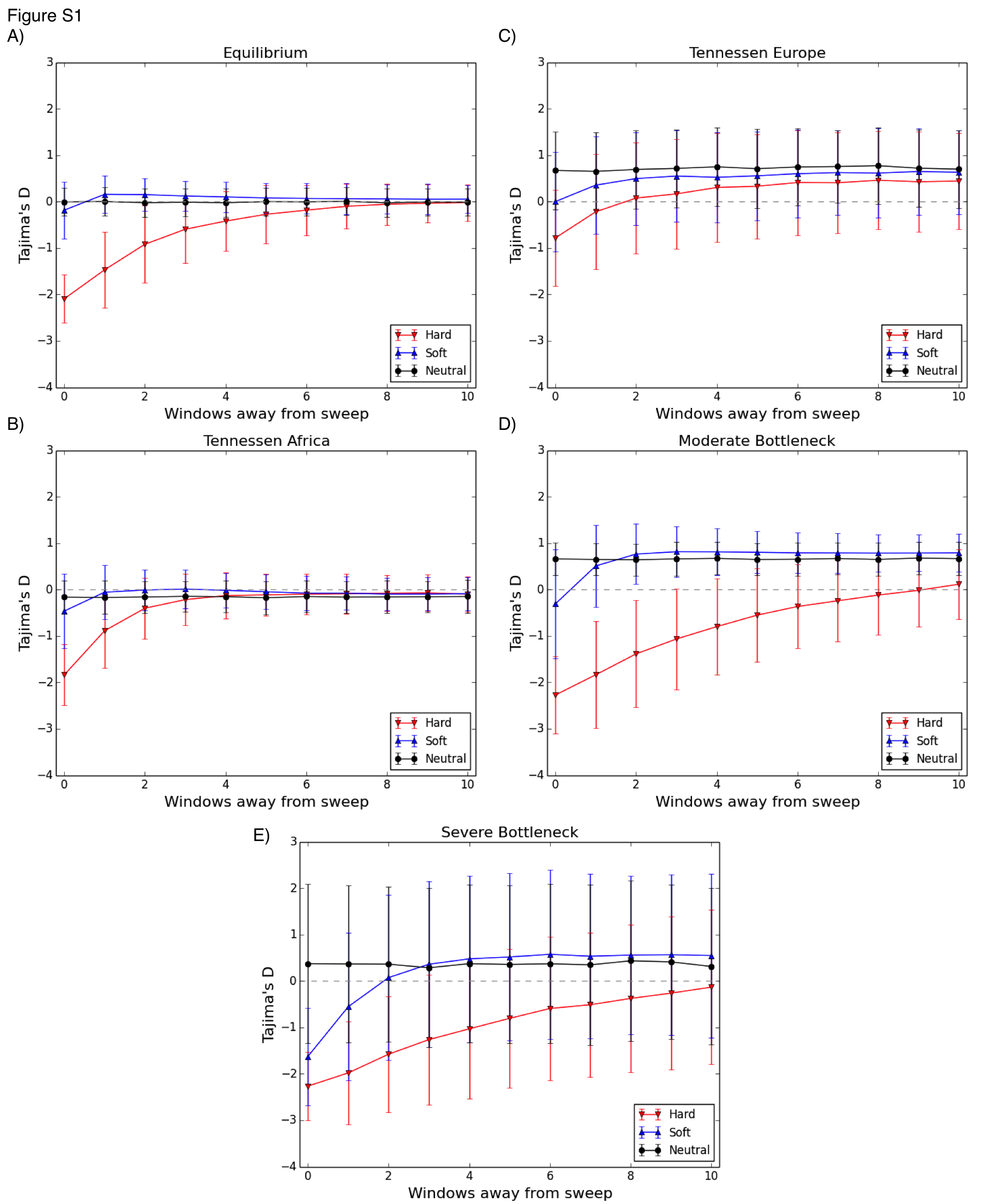

Supplement: S1 Fig — The sweep occurs in window 0. A) Values of Tajima’s D in 11 subwindows for the constant population size scenario, with α drawn from U(250, 2500). B) The African demographic model, with α drawn from U(5.0×10^3^, 5.0×10^4^). C) The European demographic model. D) The less severe bottleneck model (reduction to 29% of original size), with sweeps completing immediately prior to sampling. E) The more severe Thornton and Andolfatto [47] model (reduction to 2.9% of original size), with sweeps completing immediately prior to sampling. (TIFF) [file pgen.1005928.s001.tiff]

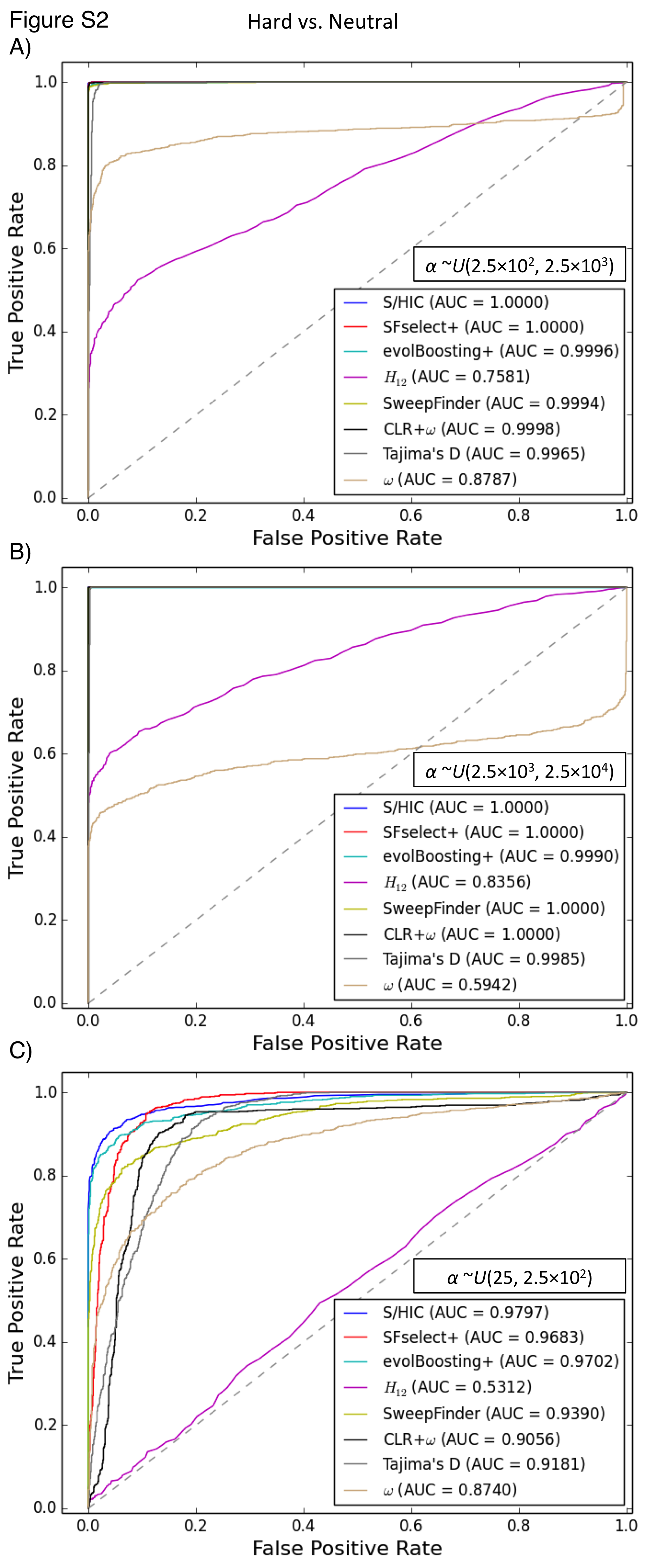

Supplement: S2 Fig — A) For intermediate strengths of selection (α~U(2.5×10^2^, 2.5×10^3^)). B) For stronger selective sweeps (α~U(2.5×10^3^, 2.5×10^4^)). C) For weaker sweeps (α~U(2.5×101, 2.5×10^2^)). Here, the methods that require training from simulated sweeps were trained from a set having the same distribution of selection coefficients as the test set. (TIFF) [file pgen.1005928.s002.tiff]

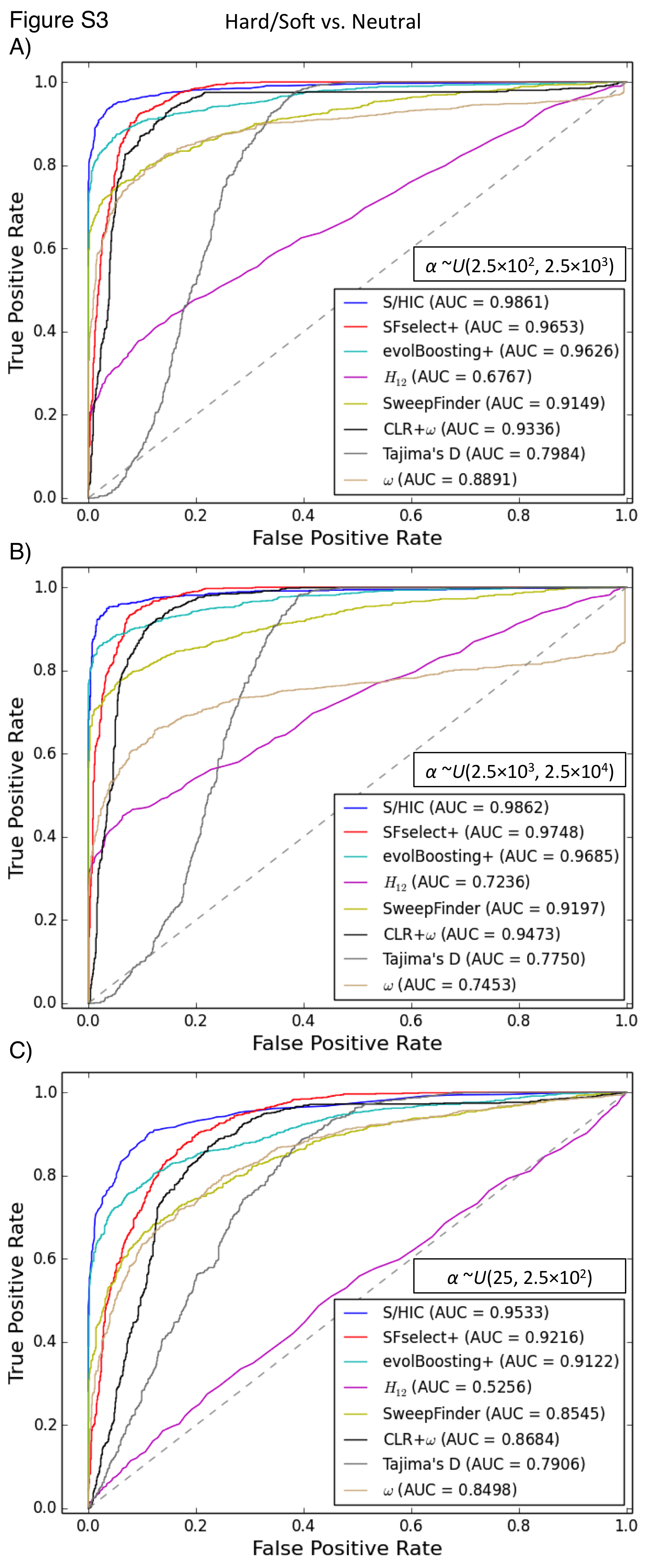

Supplement: S3 Fig — A) For intermediate strengths of selection (α~U(2.5×10^2^, 2.5×10^3^)). B) For stronger selective sweeps (α~U(2.5×10^3^, 2.5×10^4^)). C) For weaker sweeps (α~U(2.5×101, 2.5×10^2^)). (TIFF) [file pgen.1005928.s003.tiff]

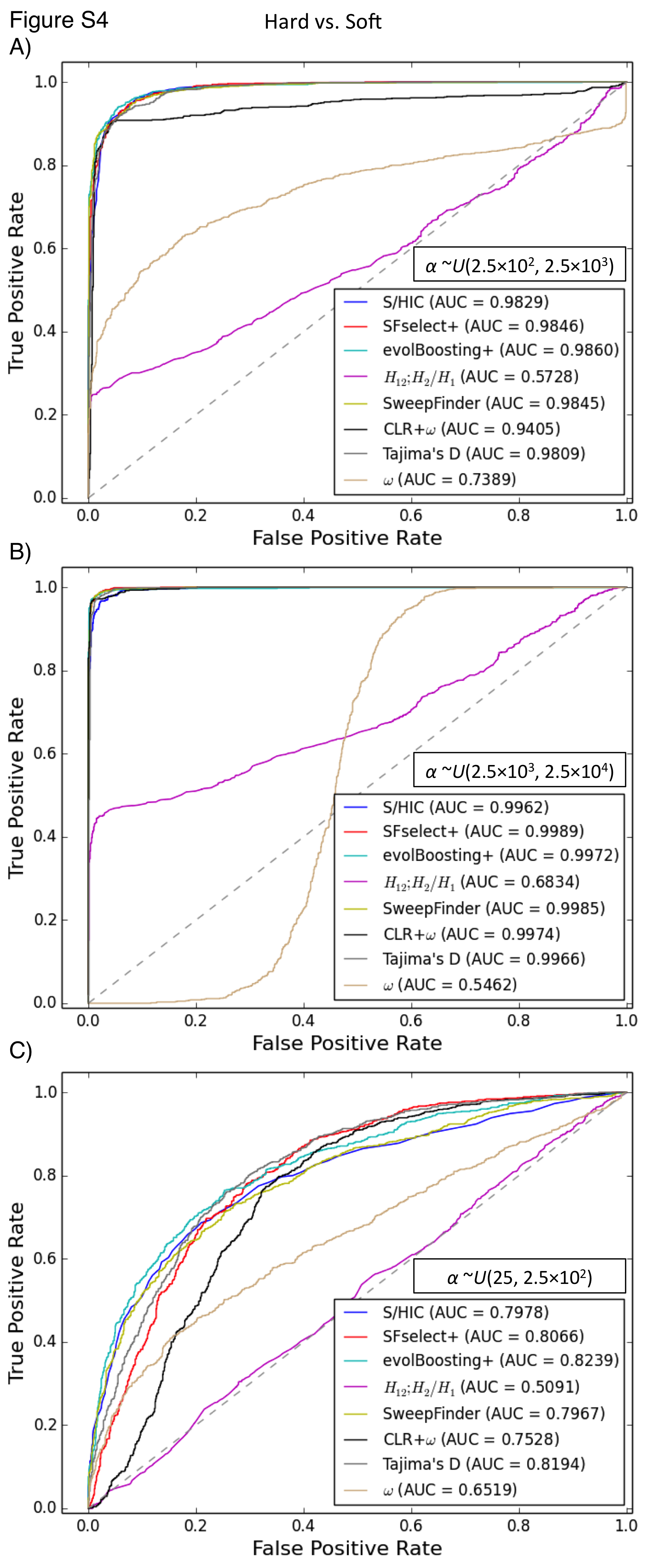

Supplement: S4 Fig — A) For intermediate strengths of selection (α~U(2.5×10^2^, 2.5×10^3^)). B) For stronger selective sweeps (α~U(2.5×10^3^, 2.5×10^4^)). C) For weaker sweeps (α~U(2.5×101, 2.5×10^2^)). (TIFF) [file pgen.1005928.s004.tiff]

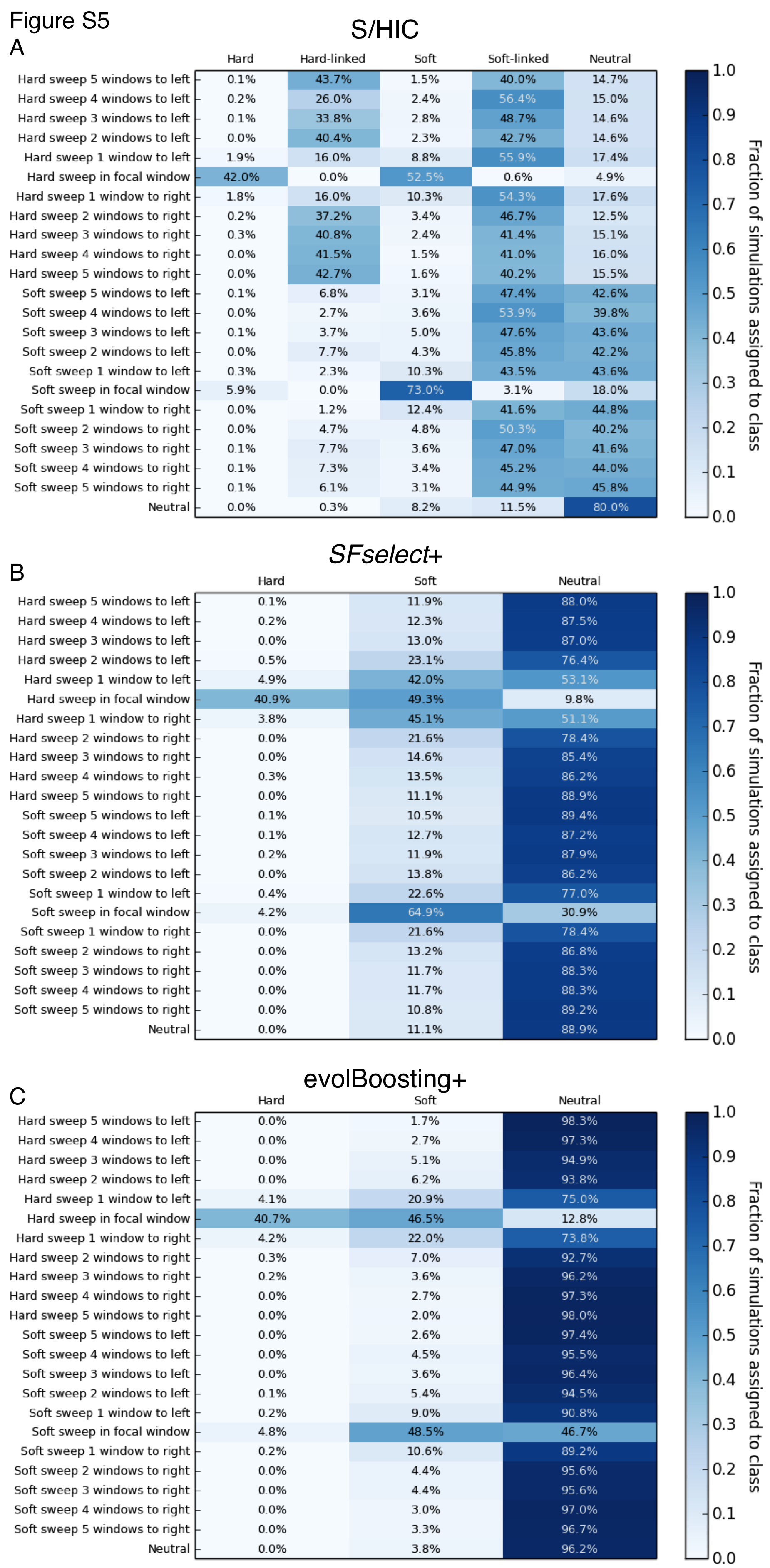

Supplement: S5 Fig — The location of any sweep relative to the classified window (or "Neutral" if there is no sweep) is shown on the y-axis, while the inferred class on the x-axis. Here, α~U(2.5×101, 2.5×10^2^). A) Results for S/HIC. B) SFselect+. C) evolBoosting+. (TIFF) [file pgen.1005928.s005.tiff]

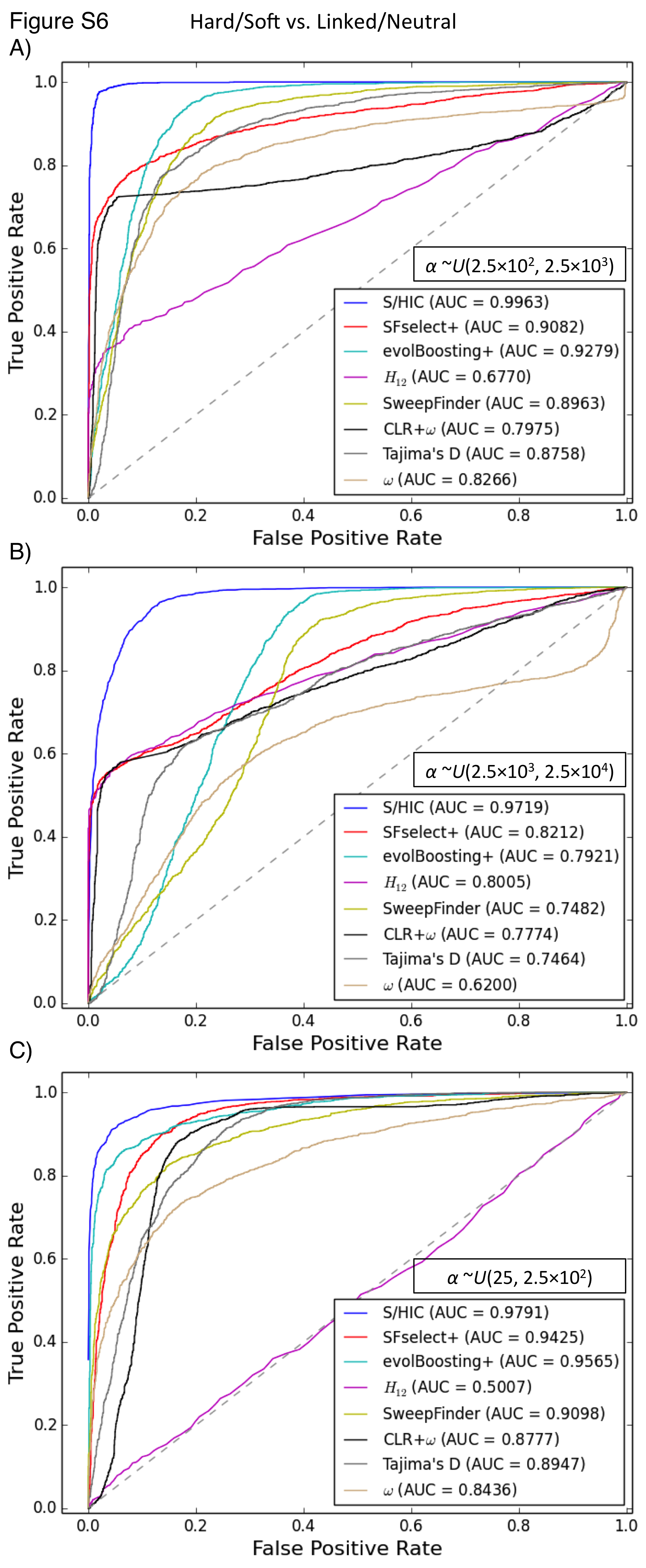

Supplement: S6 Fig — A) For intermediate strengths of selection (α~U(2.5×10^2^, 2.5×10^3^)). B) For stronger selective sweeps (α~U(2.5×10^3^, 2.5×10^4^)). C) For weaker sweeps (α~U(2.5×101, 2.5×10^2^)). For the soft sweep training and test examples used to generate these plots, f was drawn from ~U(2/2N, 0.05). (TIFF) [file pgen.1005928.s006.tiff]

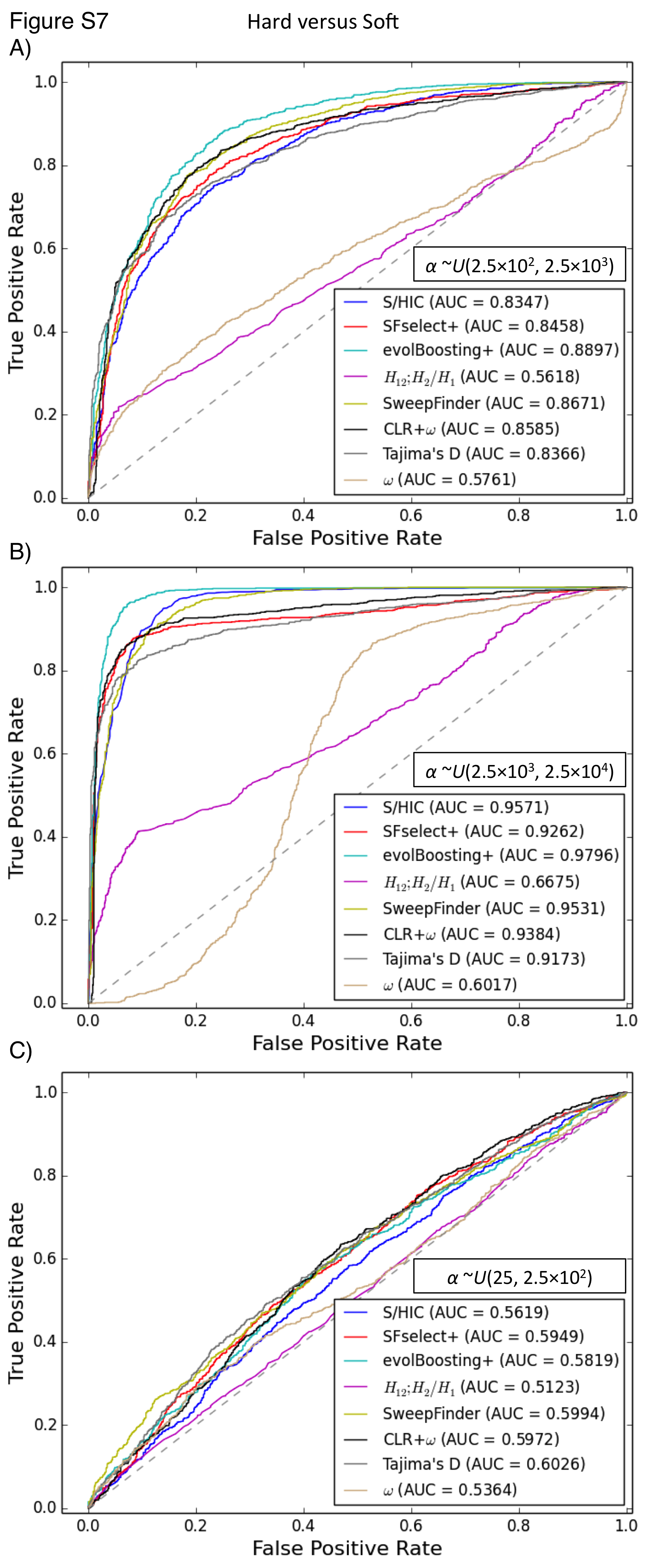

Supplement: S7 Fig — A) For intermediate strengths of selection (α~U(2.5×10^2^, 2.5×10^3^)). B) For stronger selective sweeps (α~U(2.5×10^3^, 2.5×10^4^)). C) For weaker sweeps (α~U(2.5×101, 2.5×10^2^)). For the soft sweep training and test examples used to generate these plots, f was drawn from ~U(2/2N, 0.05). (TIFF) [file pgen.1005928.s007.tiff]

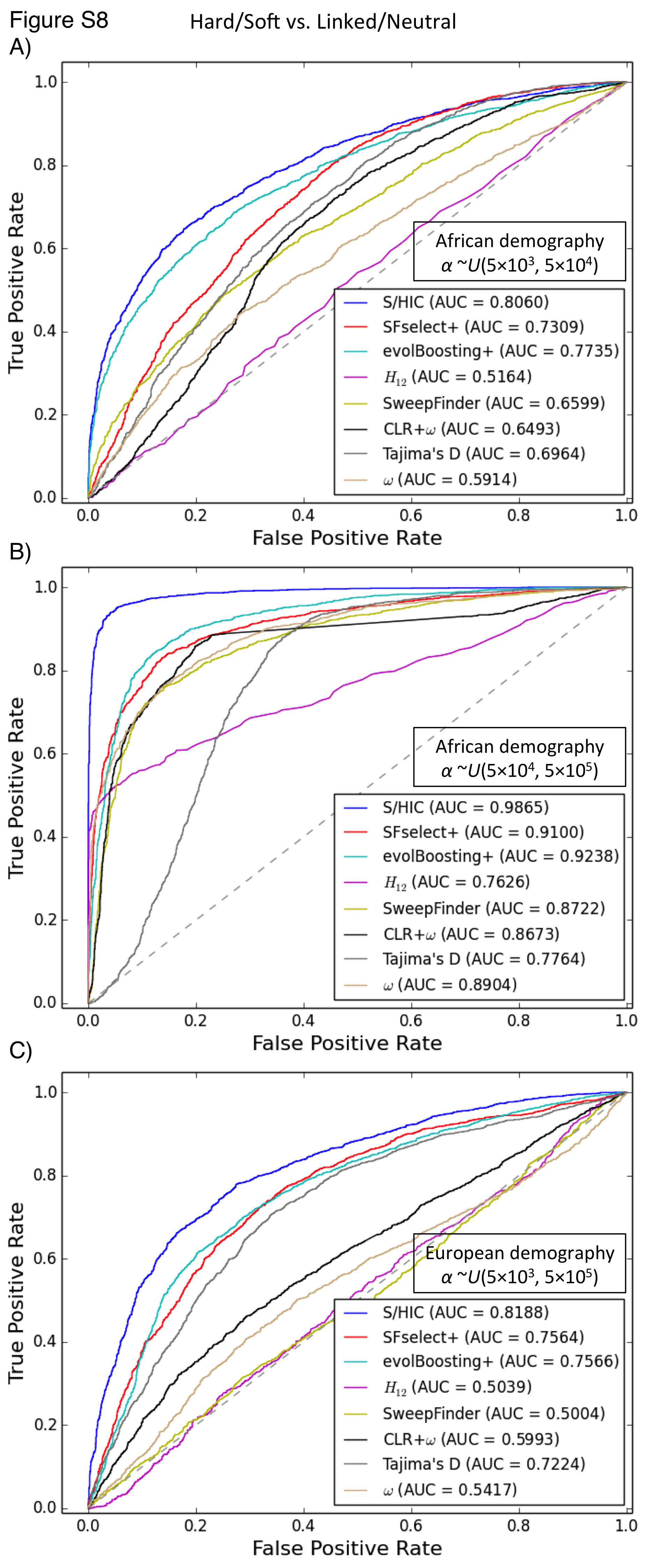

Supplement: S8 Fig — Here, the methods that require training from simulated sweeps were trained from the same demographic model used for testing. A) Testing on the African demographic model, with α~U(5×10^3^, 5×10^4^). B) The African demographic model, with α~U(5×10^4^, 5×10^5^). C) The European demographic model, with α~U(5×10^3^, 5×10^5^). (TIFF) [file pgen.1005928.s008.tiff]

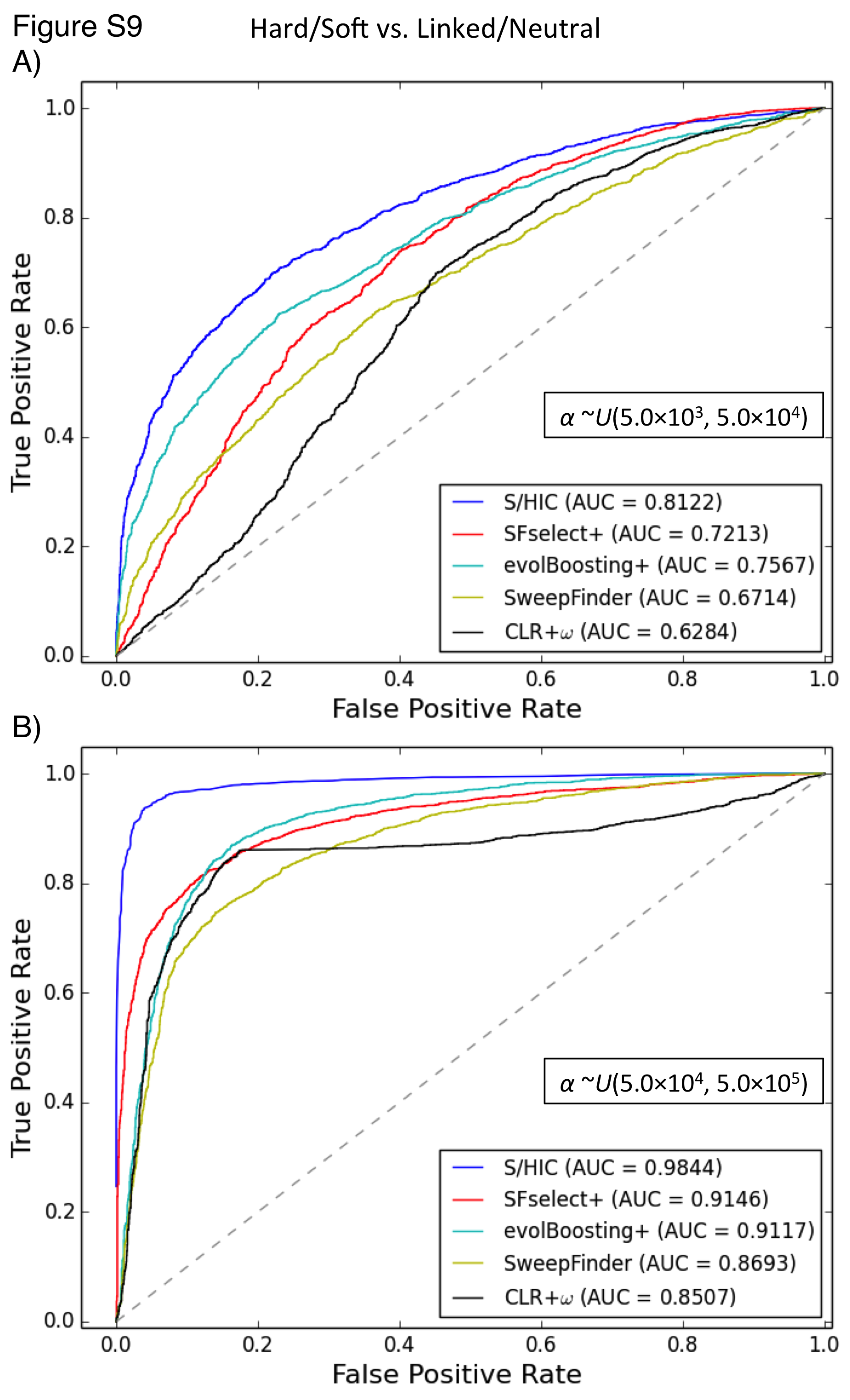

Supplement: S9 Fig — Here, the methods that require training from simulated sweeps were trained from the same simulations with equilibrium demography as used for Figs 2–5. A) Testing on the African demographic model, with α~U(5×10^3^, 5×10^4^). B) The African demographic model, with α~U(5×10^4^, 2.5×10^5^). Note that Tajima’s D and Kim and Nielsen’s ω were omitted from this figure, as we simply used the values of these statistics to generate ROC curves without respect to any demographic model. (TIFF) [file pgen.1005928.s009.tiff]

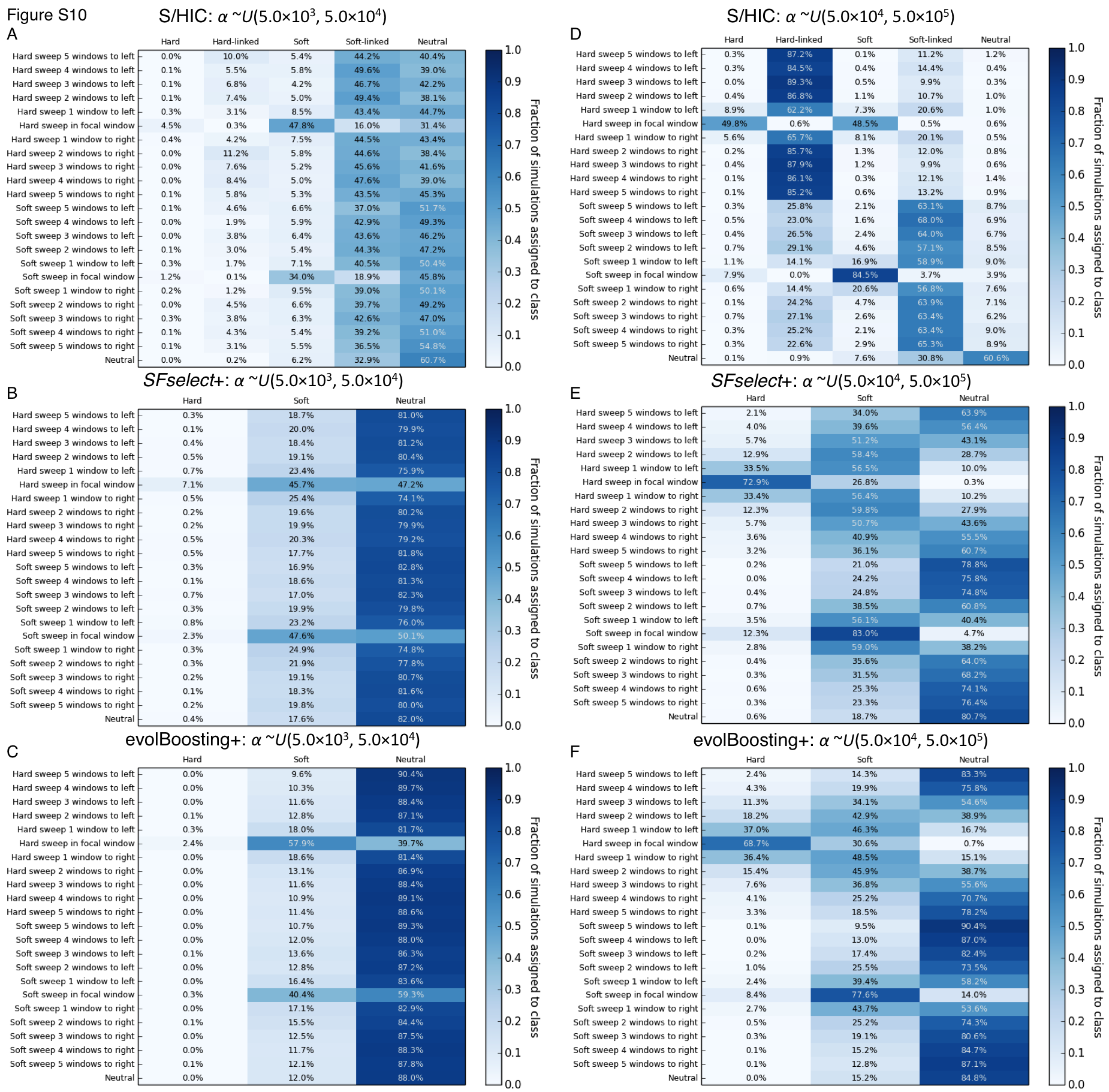

Supplement: S10 Fig — The location of any sweep relative to the classified window (or "Neutral" if there is no sweep) is shown on the y-axis, while the inferred class on the x-axis. For panels A–C, α~U(5×10^3^, 5×10^4^), and for D–F α~U(5×10^4^, 5×10^5^). These three classifiers were trained from simulations with equilibrium demography. (TIFF) [file pgen.1005928.s010.tiff]

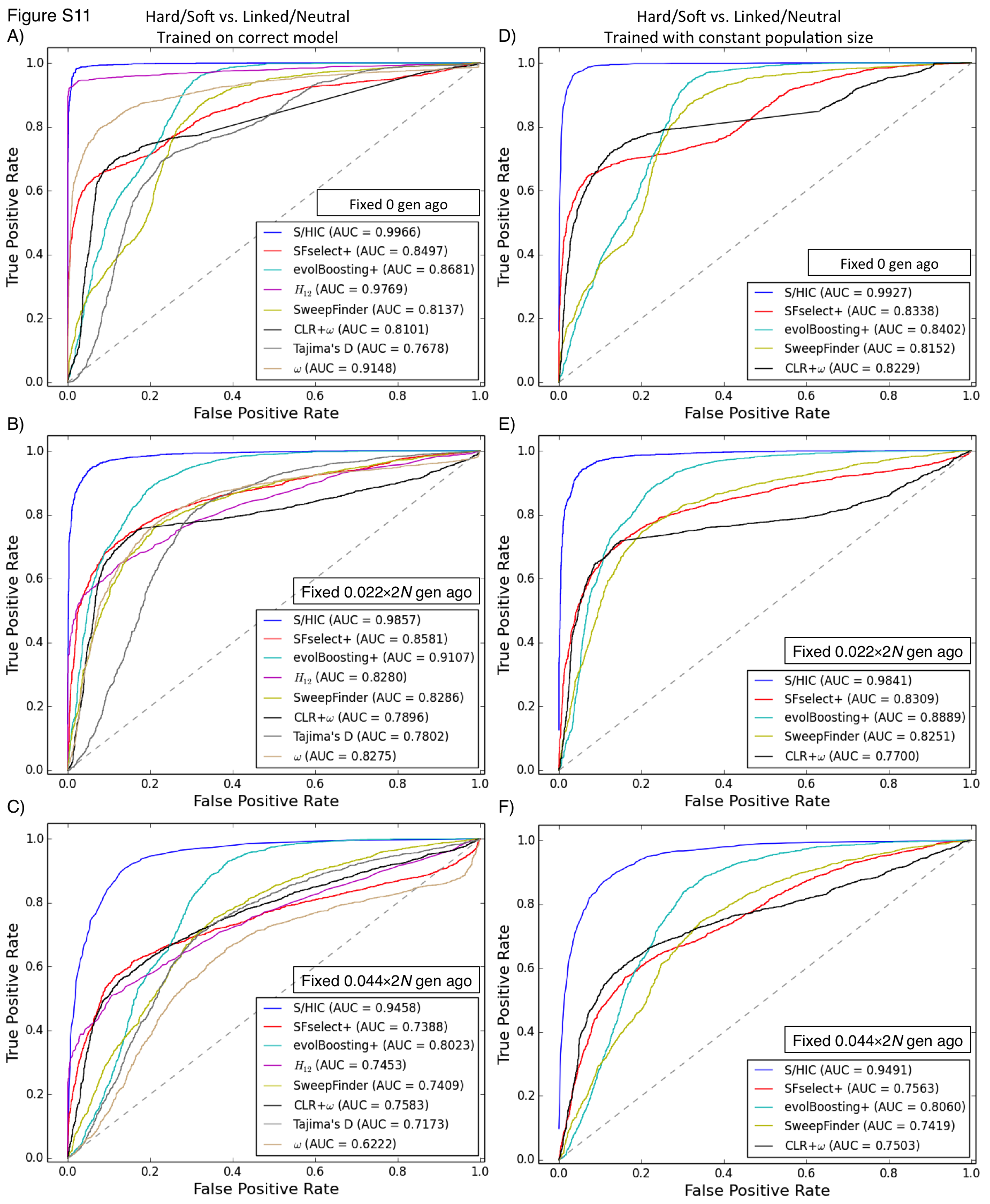

Supplement: S11 Fig — A) For very recent sweeps (fixation immediately prior to sampling). B) For older sweeps (fixation 0.22×2N generations ago). C) For the oldest sweeps (fixation 0.44×2N generations ago). D) For very recent sweeps, but after training on equilibrium demography. E) For older sweeps, after training under equilibrium. F) For the oldest sweeps, after training under equilibrium. (TIFF) [file pgen.1005928.s011.tiff]

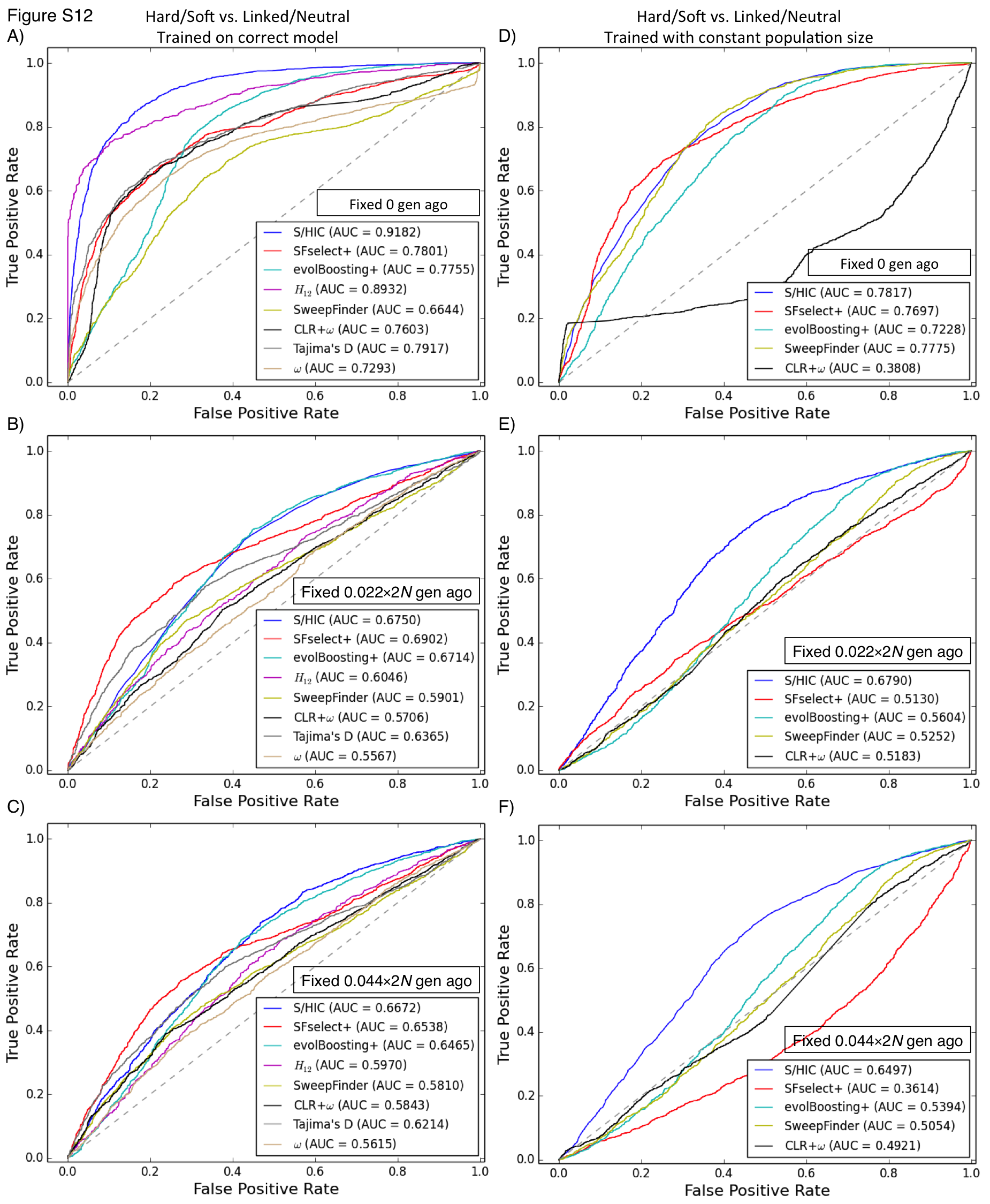

Supplement: S12 Fig — A) For very recent sweeps (fixation immediately prior to sampling). B) For older sweeps (fixation 0.22×2N generations ago). C) For the oldest sweeps (fixation 0.44×2N generations ago). D) For very recent sweeps, but after training on equilibrium demography. E) For older sweeps, after training under equilibrium. F) For the oldest sweeps, after training under equilibrium. (TIFF) [file pgen.1005928.s012.tiff]

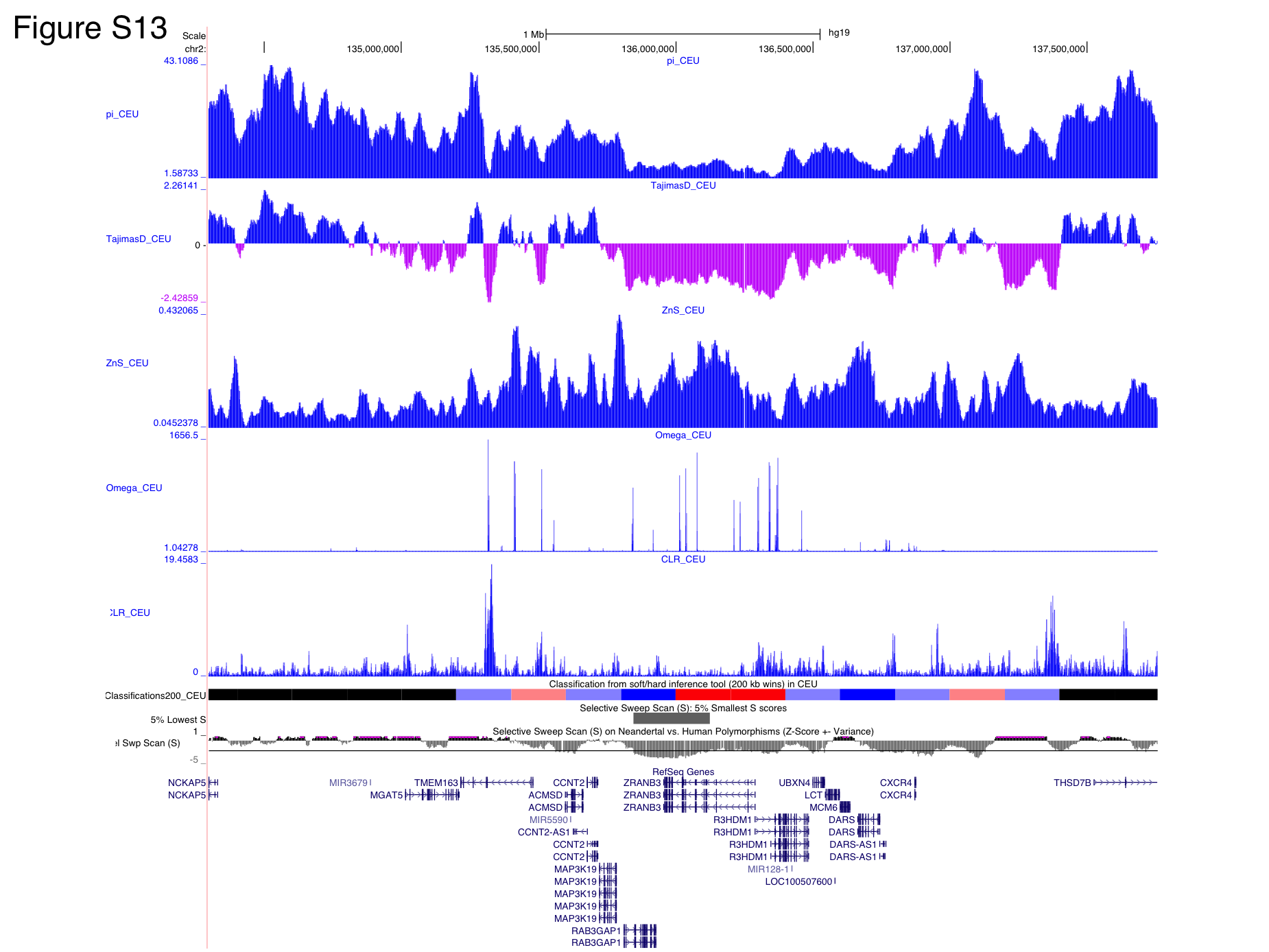

Supplement: S13 Fig — Values of π, Tajima’s D, Kelley’s ZnS, Kim and Nielsen’s ω, and Nielsen et al’s composite likelihood ratio, all from Pybus et al. [59], are shown. Beneath these statistics we show the classifications from S/HIC (red: hard sweep; faded red: hard-linked; blue: soft sweep; faded blue: soft-linked; black: neutral). This image was generated using the UCSC Genome Browser (http://genome.ucsc.edu). (TIFF) [file pgen.1005928.s013.tiff]
